# Supplementary material for: From Cohort to Cohort: A Similar Mixture Approach (SMACH) to Evaluate Exposures to a Mixture Leading to Thyroid-Mediated Neurodevelopmental Effects Using NHANES Data
Source: Toxics. 2023 Mar 31;11(4):331. doi: 10.3390/toxics11040331 (PMC10142960; doi:10.3390/toxics11040331)
Supplement: Supplementary file 1 [file toxics-11-00331-s001.zip › toxics-2284328-supplementary.pdf]

Supplementary Data: R code for HTTK and SMACH (with and without sampling weights)

```
### HTTK modeling for Mix N to be used in a SMACH with NHANES data
### Feb 13, 2023

library(httk)
library(scales)
library(tidyverse)
library(data.table)
library(EnvStats)
library(haven)
library(survey)
library(tidyr)


##### A) Toxicokinetics ####

# MIX N case chemicals
BPA <- '80-05-7'
MBzP <- '2528-16-7'
MBP <- '131-70-4'
MINP <- '106610-61-1'
MEP <- "2306-33-4"
PFHXS <- '355-46-4'
PFNA <- '375-95-1'
PFOS <-"1763-23-1"


#version control
packageVersion("httk")  >= "2.2.1"

# A chemical inventory should be loaded locally with PBK specific parameters
load_sipes2017()
load_pradeep2020(overwrite = FALSE, target.env = .GlobalEnv)
load_dawson2021(overwrite = FALSE, exclude_oad = TRUE, target.env = .GlobalEnv)


##### The loaded chemical inventory does not include parameters of MEP and MINP, but includes parameters f
#or their respective parent compounds, DEP (CAS:'84-66-2') and DINP(CAS:'28553-12-0')
# To address that, it was assumed that:
# Assumption 1: hepatic clearance of parent = hepatic clearance of metabolite
# Assumption 2: fraction unbound of parent = fraction unbound of metabolite
# pKa values were predicted using Marvin Protonation Plugin

#To add MEP and MINP parameters in the local inventory run the following:

#### MEP
my.new.data <- as.data.frame(c("Monoethyl phthalate"),stringsAsFactors=FALSE)
my.new.data <- cbind(my.new.data,as.data.frame(c("2306-33-4"),stringsAsFactors=FALSE))
my.new.data <- cbind(my.new.data,as.data.frame(c("DTXSID3052696"),stringsAsFactors=FALSE))
my.new.data <- cbind(my.new.data,as.data.frame(c(194.19)))
my.new.data <- cbind(my.new.data,as.data.frame(c(1.99)))
my.new.data <- cbind(my.new.data,as.data.frame(c(0.19)))
```

```
my.new.data <- cbind(my.new.data,as.data.frame(c(42.5)))
colnames(my.new.data) <- c("Name","CASRN","DTXSID","MW","LogP","Fup","CLint")

chem.physical_and_invitro.data <- add_chemtable(my.new.data,
                              current.table=chem.physical_and_invitro.data,
                              data.list=list(
                                Compound="Name",CAS="CASRN",DTXSID="DTXSID",
                                MW="MW",logP="LogP",Funbound.plasma="Fup",
                                Clint="CLint"),
                              species="Human",
                              reference="Marvin Partitioning Plugin")

# Initialize a column describing proton donors ("acids")
my.new.data$pka.a <- NA
# set chemical to an acid (pKa_donor):
my.new.data[my.new.data$CASRN=="2306-33-4","pka.a"] <- "3.08"
chem.physical_and_invitro.data <- add_chemtable(my.new.data,
                              current.table=chem.physical_and_invitro.data,
                              data.list=list(Compound="Name",CAS="CASRN",DTXSID="DTXSID",pKa_Donor="pka.a"),
                              species="Human",
                              reference="Marvin Protonation Plugin")

rm(my.new.data)



#### MINP: set input parameters
my.new.data <- as.data.frame(c("Monoisononyl phthalate"),stringsAsFactors=FALSE)
my.new.data <- cbind(my.new.data,as.data.frame(c('106610-61-1'),stringsAsFactors=FALSE))
my.new.data <- cbind(my.new.data,as.data.frame(c("DTXSID80910057"),stringsAsFactors=FALSE))
my.new.data <- cbind(my.new.data,as.data.frame(c(292.38)))
my.new.data <- cbind(my.new.data,as.data.frame(c(5.02)))
my.new.data <- cbind(my.new.data,as.data.frame(c(0.19)))
#### For MINP, there was limited availability of PBK parameters,
# and hepatic clearance was set equal to the reported DINP Clint as found in chem.physical_and_invitro.data
my.new.data <- cbind(my.new.data,as.data.frame(c(137.80)))
colnames(my.new.data) <- c("Name","CASRN","DTXSID","MW","LogP","Fup","CLint")

chem.physical_and_invitro.data <- add_chemtable(my.new.data,
                              current.table=chem.physical_and_invitro.data,
                              data.list=list(
                                Compound="Name",CAS="CASRN",DTXSID="DTXSID",
                                MW="MW",logP="LogP",Funbound.plasma="Fup",
                                Clint="CLint"),
                              species="Human",
                              reference="Marvin Partitioning Plugin")

# Initialize a column describing proton donors ("acids")
my.new.data$pka.a <- NA
# set chemical to an acid (pKa_donor):
my.new.data[my.new.data$CASRN=='106610-61-1',"pka.a"] <- "3.08"
chem.physical_and_invitro.data <- add_chemtable(my.new.data,
                              current.table=chem.physical_and_invitro.data,
```

```
                              data.list=list(Compound="Name",CAS="CASRN",DTXSID="DTXSID",pKa_Donor="pka.a"),
                              species="Human",
                              reference="Marvin Protonation Plugin")

rm(my.new.data)


# MIX N


mixN <- data.table(X=c(1:8),
        chem=c('MEP','MBP','MBzP','MINP','BPA','PFHxS','PFNA','PFOS'),
        matrix= c('u','u','u','u','u','p','p','p'), #matrix measured in u= urine, p=plasma
        CAS=c(MEP,MBP,MBzP,MINP,BPA,PFHXS,PFNA,PFOS),
        ref_mix=c(0.000000027,2.26E-08,1.05E-08,2.06E-08,0.000000004,3.2E-09,1.1E-09,1.03E-08),
        # mixture proportions from which PoD has been derived as found in Caporale et al. 2022
        MW=c(194.18,222.24,256.25,292.4,228.29,400.12,464.08,500.13),
        NHANES.variable=c('URXMEP','URXMBP','URXMZP','URXMNP','URXBPH','LBXPFHS','LBXPFNA','PFOS')
)



# Physiological parameters
Vurine=1.6 #in liters, Sakhi et al. 2018
dose=1
BW= 78.5 #set to representative weight for cohort


### For chemicals measured in urine:

u <- as.data.table(mixN[matrix=='u',])
output <- drop_na(data.table(CAS=NA,
            Aurine=NA,
            Ametabolised=NA,
            Cplasma=NA
))

for (CAS in u$CAS) {

  parameters <- parameterize_pbtk(chem.cas = CAS, species = 'Human')
  parameters['BW'] <- BW
  model <- solve_pbtk(chem.cas=CAS,
           parameters = parameters,
           dose=0, days=180,
           doses.per.day = 3,
           daily.dose = dose,
           plots=FALSE, output.units = 'uM'
  )



  model <- as.data.table(model)
  calc <- model[time>98.9 & time<100.1,]
```

```
  out <- data.table(CAS=CAS,
                Aurine=max(calc$Atubules)-min(calc$Atubules), # in µmol
                Ametabolised=max(calc$Ametabolized)-min(calc$Ametabolized), # in µmol
                Cplasma=median(calc$Cplasma)
  )

  output<- rbind(out,output)


  rm(parameters,model,calc,out)

}


u <- merge(u,output, by='CAS')

#### For chemicals included in the httk inventory:
u <- data.table(u,Curine=(u$Aurine*u$MW/Vurine)) # in µg/L,
#µmol in urine (Atubules) and metabolites (Ametabolised) over urine volume in 24 (L) and converted in µg (MW)
u<- data.table(u,urine_to_plasma=(u$Cplasma/u$Curine),plasma_uM_L=1)
#adding conversion factor columns for SMACH analysis
u <- u[,c(1,3:7,12:13)]

### For chemicals measured in plasma:
p <- as.data.table(mixN[matrix=='p',])
p <- data.table(p,urine_to_plasma=1, plasma_uM_L=1/p$MW)
p <- p[,c(2:9)]


# Toxicokinetics to SMACH input  ----------------------------------------------------------

SMACH <- data.table(chemID= c(1:8),rbind(u,p))

rm(p,u,output,CAS,chem.physical_and_invitro.data,mixN,BPA,MBP,MBzP,MEP,MINP,PFHXS,PFNA,PFOS,BW,dose,Vurine)

directory_path = "/Users/gennic01/Desktop/SMACH in R/"
write.csv(SMACH, paste0(directory_path, "MixN 021323.csv"))
```

```
### SMACH using NHANES data with Mixture N0 (Caporole et al 2022)

### import packages into libraries
#rm(list = ls())
#install.packages("survey")
#install.packages("haven")
#install.packages("rmarkdown")

library(survey) ## svydesigb, svymean, svglm
library(dplyr)  ## select, mutate, recode
library(haven)  ## read_xpt
library(rmarkdown)
library(ggplot2)
################################################################################
# define the path
directory_path = "/Users/gennic01/desktop/SMACH in R/"
################################################################################
# Download NHANES 2015-2016 files
PHTH_I <- read_xpt(url("https://wwwn.cdc.gov/Nchs/Nhanes/2015-2016/PHTHTE_I.XPT")) %>%
        select(SEQN, URXMEP, URXMBP, URXMZP, URXMNP, URXCOP)  ## units are ng/mL = ug/L
PHTH_H <- read_xpt(url("https://wwwn.cdc.gov/Nchs/Nhanes/2013-2014/PHTHTE_H.XPT")) %>%
        select(SEQN, URXMEP, URXMBP, URXMZP, URXMNP, URXCOP)  ## units are ng/mL = ug/L
PHTH_G <- read_xpt(url("https://wwwn.cdc.gov/Nchs/Nhanes/2011-2012/PHTHTE_G.XPT")) %>%
        select(SEQN, URXMEP, URXMBP, URXMZP, URXMNP, URXCOP)  ## units are ng/mL = ug/L
PHTH <- rbind(PHTH_I, PHTH_H, PHTH_G)   ## units are ng/mL = ug/L
dim(PHTH)

EPHPP_I <- read_xpt(url("https://wwwn.cdc.gov/Nchs/Nhanes/2015-2016/EPHPP_I.XPT")) %>% select(SEQN, URXBPH)   ##
units are ng/mL = ug/L
EPHPP_H <- read_xpt(url("https://wwwn.cdc.gov/Nchs/Nhanes/2013-2014/EPHPP_H.XPT")) %>% select(SEQN, URXBPH)
## units are ng/mL = ug/L
EPH_G <- read_xpt(url("https://wwwn.cdc.gov/Nchs/Nhanes/2011-2012/EPH_G.XPT")) %>% select(SEQN, URXBPH)   ## units
are ng/mL = ug/L
EPHPP <- rbind(EPHPP_I, EPHPP_H, EPH_G)
dim(EPHPP)


# because we combine data across 3 2yr cycles, we divide the 2yr weight by 3
# combinedhttps://wwwn.cdc.gov/nchs/nhanes/tutorials/module3.aspx
PFAS_I <- read_xpt(url("https://wwwn.cdc.gov/Nchs/Nhanes/2015-2016/PFAS_I.XPT")) %>%
        mutate(PFOS = LBXNFOS + LBXMFOS, WTS6YR = WTSB2YR/3) %>%
        select(SEQN, LBXPFHS, LBXPFNA, PFOS,  WTS6YR)
PFAS_H <- read_xpt(url("https://wwwn.cdc.gov/Nchs/Nhanes/2013-2014/PFAS_H.XPT"))
PFAS_H2 <- read_xpt(url("https://wwwn.cdc.gov/Nchs/Nhanes/2013-2014/SSPFAS_H.XPT"))
PFAS_H <- merge(PFAS_H, PFAS_H2, by="SEQN") %>%
        mutate(PFOS = SSNPFOS + SSMPFOS, WTS6YR=WTSSBH2Y/3) %>%
        select(SEQN, LBXPFHS, LBXPFNA, PFOS, WTS6YR)
## earlier cycles did not include branches of
PFAS_G <- read_xpt(url("https://wwwn.cdc.gov/Nchs/Nhanes/2011-2012/PFC_G.XPT")) %>%
        mutate(PFOS = LBXPFOS, WTS6YR=WTSA2YR/3) %>%
        select(SEQN, LBXPFHS, LBXPFNA, PFOS, WTS6YR)
```

```
names(PFAS_G)
names(PFAS_H)
names(PFAS_I)

PFAS = rbind(PFAS_I, PFAS_H, PFAS_G) ## units are ng/mL = ug/L
dim(PFAS)
summary(PFAS)

DEMO_I <- read_xpt(url("https://wwwn.cdc.gov/Nchs/Nhanes/2015-2016/DEMO_I.XPT")) %>%
    select(SEQN, RIAGENDR, RIDAGEYR, RIDEXPRG, INDFMPIR, RIDRETH1, SDMVPSU, SDMVSTRA)
DEMO_H <- read_xpt(url("https://wwwn.cdc.gov/Nchs/Nhanes/2013-2014/DEMO_H.XPT")) %>%
    select(SEQN, RIAGENDR, RIDAGEYR, RIDEXPRG, INDFMPIR, RIDRETH1, SDMVPSU, SDMVSTRA)
DEMO_G <- read_xpt(url("https://wwwn.cdc.gov/Nchs/Nhanes/2011-2012/DEMO_G.XPT")) %>%
    select(SEQN, RIAGENDR, RIDAGEYR, RIDEXPRG, INDFMPIR, RIDRETH1, SDMVPSU, SDMVSTRA)
DEMO = rbind(DEMO_I, DEMO_H, DEMO_G)
DEMO <- DEMO %>% mutate(sex = recode(RIAGENDR, '1'="M", '2' = "F"))
DEMO$pregnant = ifelse(DEMO$RIDEXPRG==1, 1,0)
DEMO$pregnant[is.na(DEMO$pregnant)] = 0
table(DEMO$RIDEXPRG)
table(DEMO$pregnant)
table(DEMO$sex)

DEMO$race_eth <- factor(DEMO$RIDRETH1,
        level=c(1,2,3,4,5),
        labels=c("MexAmer", "OtherHisp", "NonHispWhite", "NonHispBlack", "Other/MultiRace"))
DEMO$race_eth <- relevel(DEMO$race_eth, ref=3)
DEMO$race_eth_bin = (DEMO$RIDRETH1 !=3)
table(DEMO$RIDRETH1)
table(DEMO$race_eth, DEMO$race_eth_bin)

# combine into a single dataset
concdata = merge(PHTH, EPHPP, by="SEQN")
concdata = merge(concdata, PFAS, by="SEQN")
concdata = merge(concdata, DEMO, by="SEQN")

names(concdata)
dim(concdata)
head(concdata)


## do not subset yet since we need svy wts to be calculated for correct subset
#concdata_F <- concdata  %>% filter(sex=="F" & 15<RIDAGEYR & RIDAGEYR<46)
dim(concdata)
write.csv(concdata, paste0(directory_path, "NHANESconcdata.csv"))
########################################################################################
## read in mixture characteristics data with variable names that match the mixture names
mixN = read.csv(paste0(directory_path, "MixN 021323.csv"))
mixN
########################################################################################
mixN$chem
mixN$ref_mix ###### reference mixture (mols/L) ## this is MIXN from Paris slides
sum_ref_mix = sum(mixN$ref_mix)
sum_ref_mix
```

```
## create name vectors
chems = mixN$NHANES.variable   # chemicals all in units of ug/L in order of ref_mix
#chems = c("URXMEP", "URXMBP", "URXMZP", "URXMNP", "URXBPH", "LBXPFHS", "LBXPFNA", "PFOS")
chems

### create names for Cp values
Cp <- paste0(chems, "_CP")
Cp


# estimate uM in plasma for each chem
concdata[, Cp] = as.matrix(concdata[, chems]) %*% diag(mixN$urine_to_plasma*mixN$plasma_uM_L)  ## translate to Cp uM
concdata[, Cp] = concdata[, Cp]/(10**6)  ## translate to Cp in mols/L

# create mixing proportions from ref mixture and each candidate (made into complete case)
sum_ref_mix = sum(mixN$ref_mix)
ref_prop = mixN$ref_mix/sum_ref_mix
round(ref_prop, 4)

Cp_prop = (concdata[,Cp]/rowSums(concdata[,Cp]))
Cp_prop = Cp_prop[complete.cases(Cp_prop[,Cp]),]  ## does using complete case change the sampling weights?
dim(Cp_prop)
round(colMeans(Cp_prop),4)
geom_cp = exp(colMeans(log(Cp_prop)))
geom_cp
check = geom_cp/sum(geom_cp)
check


## BMD from XETA Caporale et al 2022 Table S7 on log10(conc+1) scale where conc=1X
BMD_est = 1.16  # in 1X units
BMD_est_conc = (10^(BMD_est)-1)*sum_ref_mix  # in mols/L
SE_BMDest = 0.13
BMDL = 0.95
BMDL_conc = (10^(BMDL)-1)*sum_ref_mix
SufSim_radius = 0.89

### calculate distance and SE(distance)
diff = Cp_prop - ref_prop
head(diff)
dim(diff)
ssq_diff = as.matrix(diff)%*%t(as.matrix(diff))
dim(ssq_diff)
is.matrix(ssq_diff)
distance = BMD_est*sqrt(diag(ssq_diff))
summary(distance)
SE_dist = SE_BMDest*distance/BMD_est

## create upper conf limit on distance
df = nrow(as.matrix(distance))
tcrit = qt(0.95, df)
upper1sidedConfLimit = distance + SE_dist*tcrit
```

```
summary(upper1sidedConfLimit)
suffsim = (upper1sidedConfLimit < SufSim_radius)

concdata = concdata[complete.cases(concdata[, Cp]), ]
concdata$suffsim = suffsim
sum(suffsim)/df
table(suffsim)

## calculate SMRI values to be used with the sampling weights below
# SMRI_HI_ref is (total concentration per subject divided by BMDL) * num of chems
# and assumes obs proportions are equivalent to ref mixture
concdata$SMRI_HI_ref =  rowSums(as.matrix(concdata[, Cp]))*ncol(as.matrix(concdata[, Cp]))/BMDL_conc
# SMRI_HI sums the estimated relative proportions of observed and ref concentrations)
concdata$SMRI_HI =  rowSums(concdata[, Cp]/(ref_prop*BMDL_conc))
concdata$SMRI_ref_GT_1 = (concdata$SMRI_HI_ref>1)
concdata$SMRI_HI_GT_1 = (concdata$SMRI_HI>1)

##############################################################################
# Survey Weights #
##############################################################################

# Here we use "svydesign" to assign the weights. We will use this new design
# variable "nhanesDesign" when running our analyses.
names(concdata)
table(concdata$sex)
allnhanesDesign <- svydesign(id   = ~SDMVPSU,
                      strata  = ~SDMVSTRA,
                      weights = ~WTS6YR,
                      nest    = TRUE,
                      data    = concdata)

svytotal(~sex, allnhanesDesign, na.rm=TRUE)
123556200 + 118215364
svyquantile(~URXMEP, allnhanesDesign, quantiles=c(0.5, 0.95, 0.99), na.rm=TRUE)
svyquantile(~URXMBP, allnhanesDesign, quantiles=c(0.5, 0.95, 0.99), na.rm=TRUE)
svyquantile(~URXMZP, allnhanesDesign, quantiles=c(0.5, 0.95, 0.99), na.rm=TRUE)
svyquantile(~URXMNP, allnhanesDesign, quantiles=c(0.5, 0.95, 0.99), na.rm=TRUE)
svyquantile(~URXBPH, allnhanesDesign, quantiles=c(0.5, 0.95, 0.99), na.rm=TRUE)
svyquantile(~LBXPFHS, allnhanesDesign, quantiles=c(0.5, 0.95, 0.99), na.rm=TRUE)
svyquantile(~LBXPFNA, allnhanesDesign, quantiles=c(0.5, 0.95, 0.99), na.rm=TRUE)
svyquantile(~PFOS, allnhanesDesign, quantiles=c(0.5, 0.95, 0.99), na.rm=TRUE)



# Here we use "subset" to tell "nhanesDesign" that we want to only look at females of reproductive age
nhanesDesignF <- subset(allnhanesDesign, (sex=="F" & 15<RIDAGEYR & RIDAGEYR<46))

svytotal(~suffsim, nhanesDesignF, na.rm=TRUE)
19838292 + 37909951
37909951/(19838292 + 37909951)


svyquantile(~URXMEP, nhanesDesignF, quantiles=c(0.5, 0.95, 0.99), na.rm=TRUE)
```

```
svyquantile(~URXMBP, nhanesDesignF, quantiles=c(0.5, 0.95, 0.99), na.rm=TRUE)
svyquantile(~URXMZP, nhanesDesignF, quantiles=c(0.5, 0.95, 0.99), na.rm=TRUE)
svyquantile(~URXMNP, nhanesDesignF, quantiles=c(0.5, 0.95, 0.99), na.rm=TRUE)
svyquantile(~URXBPH, nhanesDesignF, quantiles=c(0.5, 0.95, 0.99), na.rm=TRUE)
svyquantile(~LBXPFHS, nhanesDesignF, quantiles=c(0.5, 0.95, 0.99), na.rm=TRUE)
svyquantile(~LBXPFNA, nhanesDesignF, quantiles=c(0.5, 0.95, 0.99), na.rm=TRUE)
svyquantile(~PFOS, nhanesDesignF, quantiles=c(0.5, 0.95, 0.99), na.rm=TRUE)

# Here we use "subset" to tell "nhanesDesign" that we want to only look at a
# specific subpopulation (i.e., those with suffsim=1).
suffsimDesign <- subset(nhanesDesignF, suffsim==1)
svytotal(~suffsim, suffsimDesign)
37909951
##############################################################################
# Statistics #
##############################################################################
# We will use "svymean" to calculate the population mean for SMRI_HI_ref. The na.rm
# argument "TRUE" excludes missing values from the calculation.

svymean(~SMRI_HI_ref, suffsimDesign, na.rm = TRUE)

smrihist <- svyhist(~SMRI_HI_ref, suffsimDesign, main=NULL,
    xlab="SMRI, Survey weighted",col="darkgreen",
    breaks=30)
smrihist

svyquantile(~SMRI_HI_ref, suffsimDesign, quantiles=c(0.5, 0.9, 0.95, 0.99, 0.999), na.rm = TRUE)

svymean(~SMRI_ref_GT_1, suffsimDesign, na.rm=TRUE)
37909951*(0)



##############################################################################
# We will use "svymean" to calculate the population mean for SMRI_HI. The na.rm
# argument "TRUE" excludes missing values from the calculation.

svymean(~SMRI_HI, suffsimDesign, na.rm = TRUE)

smrihistHI <- svyhist(~SMRI_HI, suffsimDesign, main=NULL,
            xlab="SMRI, Survey weighted",col="darkgreen",
            breaks=30)
smrihistHI

svyquantile(~SMRI_HI, suffsimDesign, quantiles=c(0.5, 0.9, 0.95, 0.99, 0.999), na.rm = TRUE)

svymean(~SMRI_HI_GT_1, suffsimDesign, na.rm=TRUE)
37909951*(0.028391)
########################################
hist(log(suffsimDesign$variables$SMRI_HI))
results = svyglm(log(SMRI_HI) ~ RIDAGEYR  + log(INDFMPIR+1) + as.factor(race_eth), suffsimDesign,
          family=gaussian())
summary(results)
```

```
resultsbin = svyglm(as.factor(SMRI_HI_GT_1) ~ RIDAGEYR + log(INDFMPIR+1) + as.factor(race_eth), suffsimDesign,
                family=binomial())

resultsbin = svyglm(as.factor(SMRI_HI_GT_1) ~ RIDAGEYR + log(INDFMPIR+1) + as.factor(race_eth_bin), suffsimDesign,
                family=binomial())
summary(resultsbin)
view(print(summary(resultsbin)))

racecheck <- concdata %>% filter(suffsim==1, RIAGENDR==2, RIDAGEYR>15, RIDAGEYR<46) %>%
                     mutate(race_eth_bin = (RIDRETH1 !=3))
summary(racecheck)
table(racecheck$race_eth_bin, racecheck$SMRI_HI_GT_1)
```

```
##########################################################################
### SMACH with Mixture N0 (Caporole et al 2022) using NHANES as cohort data – that is, without sampling weights

### import packages into libraries
#rm(list = ls())
#install.packages("lazy.mat")  ## not available for R version 3.6.1 - what should I use
#install.packages("survey")
#install.packages("haven")
#install.packages("rmarkdown")

library(survey) ## svydesigb, svymean, svglm
library(dplyr)  ## select, mutate, recode
library(haven)  ## read_xpt
library(rmarkdown)
library(ggplot2)
#################################################################################
# define the path
directory_path = "/Users/gennic01/desktop/SMACH in R/"

## read in cohort data
concdata = read.csv(paste0(directory_path, "NHANESconcdata.csv"))
dim(concdata)
names(concdata)

## subset to women of reproductive age
concdata_F <- concdata %>% filter(sex=="F" & 15<RIDAGEYR & RIDAGEYR<46)
dim(concdata_F)

##############################################################
## read in mixture characteristics data
mixN = read.csv(paste0(directory_path, "mixN v2.csv"))
mixN
##############################################################
mixN$chem
mixN$ref_mix ###### reference mixture (mols/L) ## this is MIXN from Paris slides
sum_ref_mix = sum(mixN$ref_mix)
sum_ref_mix

## create name vectors
chems = mixN$NHANES_name   # chemicals all in units of ug/L in order of ref_mix
chems

mixN
### create names for Cp values
Cp <- paste0(chems, "_CP")
Cp


# estimate uM in plasma for each chem
concdata_F[, Cp] = as.matrix(concdata_F[, chems]) %*% diag(mixN$urine_to_plasma*mixN$plasma_uM_L)  ## translate to Cp
uM
concdata_F[, Cp] = concdata_F[, Cp]/(10**6)  ## translate to Cp in mols/L
```

```
# create mixing proportions from ref mixture and each candidate (made into complete case)
ref_prop = mixN$ref_mix/sum(mixN$ref_mix)
ref_prop
dim(concdata_F[,Cp])
Cp_prop = (concdata_F[,Cp]/rowSums(concdata_F[,Cp]))
Cp_prop = Cp_prop[complete.cases(Cp_prop[,Cp]),]  ## does using complete case change the sampling weights?
dim(Cp_prop)
round(colMeans(Cp_prop),4)
geom_cp = exp(colMeans(log(Cp_prop)))
geom_cp
check = geom_cp/sum(geom_cp)
check


## BMD from XETA Caporale et al 2022 Table S7 on log10(conc+1) scale where conc=1X
BMD_est = 1.16  # in 1X units
BMD_est_conc = (10^(BMD_est)-1)*sum_ref_mix  # in mols/L
SE_BMDest = 0.13
BMDL = 0.95
BMDL_conc = (10^(BMDL)-1)*sum_ref_mix
SufSim_radius = 0.89

diff = Cp_prop - ref_prop
head(diff)
dim(diff)
ssq_diff = as.matrix(diff)%*%t(as.matrix(diff))
dim(ssq_diff)
is.matrix(ssq_diff)
distance = BMD_est*sqrt(diag(ssq_diff))
summary(distance)
SE_dist = SE_BMDest*distance/BMD_est

## create upper conf limit on distance
df = nrow(as.matrix(distance))
tcrit = qt(0.95, df)
upper1sidedConfLimit = distance + SE_dist*tcrit
summary(upper1sidedConfLimit)
suffsim = (upper1sidedConfLimit < SufSim_radius)

concdata_F = concdata_F[complete.cases(concdata_F[, Cp]), ]
concdata_F$suffsim = suffsim
sum(suffsim)/df
table(suffsim)
1016/(1016+317)
## subset concdata to only those suff sim
suffsim_set <- concdata_F %>% filter(suffsim==1)
dim(suffsim_set)
summary(suffsim_set)

# SMRI_HI_ref is (total concentration per subject divided by BMDL) * num of chems
# and assumes obs proportions are equivalent to ref mixture
suffsim_set$SMRI_HI_ref =  rowSums(as.matrix(suffsim_set[, Cp]))*ncol(as.matrix(suffsim_set[, Cp]))/BMDL_conc
table(suffsim_set$SMRI_HI_ref>1)
```

```
# SMRI_HI sums the estimated relative proportions of observed and ref concentrations)
suffsim_set$SMRI_HI =  rowSums(suffsim_set[, Cp]/(ref_prop*BMDL_conc))
head(suffsim_set[, Cp]/(ref_prop*BMDL_conc))
table(suffsim_set$SMRI_HI>1)
145/(871+145)
summary(suffsim_set[, Cp])
ref_prop*BMDL_conc

summary(suffsim_set$SMRI_HI)
summary(suffsim_set$SMRI_HI_ref)
hist(suffsim_set$SMRI_HI, breaks=50)

quantile(suffsim_set$SMRI_HI_ref, probs=c(0.6, 0.7, 0.8, 0.9, 0.95, 0.99, 0.999))
suffsim_set$SMRI_HI_ref_GT_1 = (suffsim_set$SMRI_HI_ref>1)
table(suffsim_set$SMRI_HI_ref_GT_1)
quantile(suffsim_set$SMRI_HI, probs=c(0.6, 0.7, 0.8, 0.9, 0.95, 0.99, 0.999))
suffsim_set$SMRI_HI_GT_1 = (suffsim_set$SMRI_HI>1)
table(suffsim_set$SMRI_HI_GT_1)
145/(871+145)
names(suffsim_set)

smrihist <- ggplot(suffsim_set, aes(x=SMRI_HI))+
  geom_histogram(color="darkblue", fill="lightblue") +
  xlab("SMRI (Sufficiently Similar Group)") +
  geom_vline(xintercept = 1)+
  theme(axis.title=element_text(size=15))

smrihist


resultsbin = glm(as.factor(SMRI_HI_GT_1) ~ RIDAGEYR + log(INDFMPIR+1) + as.factor(race_eth), data=suffsim_set,
                 family=binomial())
summary(resultsbin)
```
